# Surgery

**Published:** 1877-06

**Authors:** 


### I. Surgery.

*Operative Treatment of Varicose Veins.* SCHEDE. (*Wiener Med. Zeitung*, 1877. No. 8.)

In the past year the writer accomplished in ten patients the artificial obliteration of the varicose veins of the leg, by ligations, in the following manner: After a bandage was applied around the thigh sufficiently firmly to make the varices swell up, the latter were exposed by an incision, carefully isolated and ligated with catgut, at the distal and proximal ends. Then the veins were cut through, between both ligatures, and the cutaneous wound was closed by interrupted silk sutures. The whole operation was performed under the carbolized spray, and an antiseptic dressing was applied afterwards, The limb thus operated upon was placed securely on a splint, in order to prevent any motion, and to obviate the breaking off of thrombi and their introduction into the circulation. The first dressing was, as a rule, not changed till the fifth day, when the sutures were removed. Although then the wound was healed, the patient was not allowed to get up till the fifteenth day. The result of this treatment was perfect, concerning the complete absence of dangerous complications and of violent reaction.

Lately the doctor has simplified his method by substituting the subcutaneous ligature. Where the vein is to be obliterated, a prepared catgut is passed around it, with a curved needle, without any cutaneous incision. The desired number of ligatures thus put in place, a piece of strong rubber tubing, of about the thickness of the little finger, is put on the skin, following the course of the vein operated upon. The ligatures are tied over this tubing, while an assistant is compressing the latter. The elastic tension of the rubber suffices to keep the ligatures perfectly tight and the opposite walls of the vein in permanent apposition. After twelve hours, one half of the ligature is removed, and after twenty-four hours, the balance. By this time, the rubber tube has made a deep groove into the skin, and is kept in its place by an antiseptic bandage, at least one week. After this period, the cure can be considered as completed. As a remarkable fact is mentioned the total absence of any decubitus, though the skin has been so long submitted to continued pressure by the rubber tubing.

*Treatment of Fractures of the Elbow in Children.* BERTHOMIER. (*Thése de Paris*, 1875; *Bullet. gén. de Thérapeut. Med. et Chir.*)

In the treatment of these injuries in children, should the member be immovably fixed in a state of flexion or extension?

According to Berthomier and Laroyenne, that which is most to be dreaded is not traumatic arthritis (which is rarely so severe as to terminate in anchylosis), but a vicious position of the fragments which in most cases causes the obstacle to natural movements. This fact has been verified in a large number of cases.

Now, proceeding from the fact that the sole position capable of bringing about exact coaptation of the fragments is that of extension, these authors have for several years treated all fractures of the elbow in children in this position. In all cases they have observed, after consolidation has been effected in these favorable conditions, that the articular stiffness yielded to appropriate treatment in about a fortnight (sometimes less), so that the joint could be placed in nearly all, if not quite all, of its normal positions. They take care to add that in some instances the indication is quite the reverse of that stated, when, for example, there might be a fear of complications due to dysthetic processes, white swelling of scrofulous subjects, etc.

The rare accident of epiphyseal separation of the epicondyle, necessitates immobility in the flexed position.

*Treatment of Carbuncle.* J. H. Dibrell, Jr., M. D. (*Phil. Med. and Surg. Reporter.*)

By the use of Carbolic acid and Collodion, good results are secured. They can be used in any stage of the disorder. D. tells us how he used these agents:—

My plan is as follows:—When the carbuncle is seen early, to puncture it, and with a camel's hair pencil, or small pointed stick, introduce into the opening thus made the pure and undiluted acid. If the disease has made greater progress, and one or more small acne-like pustules have made their appearance on the tumor, these are carefully opened, which can be done without causing pain, and the acid introduced at each opening, as before indicated. The effect of the acid when first applied, especially if it touch a denuded surface, is to produce a sharp stinging pain, which is, however, of but momentary duration. The next effect is local anæsthesia,and the patient is, for a time, perhaps hours, free from pain.

Carbolic acid possessing in a notable degree anæsthetic, antiseptic and caustic properties, would seem to be peculiarly adapted to the treatment of the disease under consideration, which is usually attended with great pain, sloughing, and an intolerable odor. Its use in my hands has certainly seemed to diminish the pain, correct the odor and to arrest the sloughing process with much promptitude.

After the acid had been applied, collodion should be several times painted over the carbuncle, and beyond it, a few lines, on the uninflamed skin. *All the openings are to be left free*, in order to give egress to discharges. Each layer or film of the collodion should be allowed to dry before another is put on. This dressing may be renewed once daily, and the collodion previously applied, if partially detached, should be peeled off before a new application is made. If the part on which the carbuncle makes its appearance be covered with hair, this should be cleanly shaved off, otherwise the collodion will be difficult to remove, and at the same time cause considerable pain.

It is interesting to watch the collodion as it contracts upon the diseased tissues. The skin, previously red and swollen, will in a few minutes be seen through transparent gun cotton, to have become pale and depressed, as the pressure gradually empties the engorged capillaries. If the disease is advanced, and sloughs have become partly separated, they are not unfrequently forced out, or brought so near the openings as to be readily detached with scissors. This pressure does not give rise to pain, but on the contrary, generally affords much relief to the suffering patient. The application of collodion in this disease has other advantages. It limits the extent of the disease in decreasing the vascularity of the part, and in this way lessens the inflammatory action going on, and probably also prevents the absorption of pus. It also protects the surronnding skin from contact with the discharges, which, as is well known, are capable of producing, if not an extension of the disease, numerous small boils, which are of themselves an exceedingly annoying complication. Should, however, any such pustules or boils be formed in the course of the disease, they can be cut short by touching them with carbolic acid. After the carbuncle has been treated with the acid and collodion, it should be protected from contact with the clothing, by covering it over with a piece of old linen or cotton cloth, saturated with sweet oil, or spread with carbolic acid cerate.

*Constant Irrigation in Chronic Cystitis.* JACKSON. (*The Boston Med. and Surg. Jour.*, April, 1877.)

Dr. J. reports two cases of chronic cystitis successfully treated by constant irrigation. The means used were a vessel containing water, a double catheter, and india-rubber tubing sufficient to convey the water to and from the bladder. The flow was regulated by a stop-cock attached to the reservoir. The position of the vessel should be such as not to cause pain by excessive pressure, but it is necessary that the bladder should be fully distended at times, in order that the whole surface may be thoroughly cleansed. About a barrel of water is needed in twenty-four hours. Of the first case, he says that, the usual method of intermittent irrigation was adopted, and continued about two months, without benefiting the patient, at the expiration of which time constant irrigation day and night by means of water about the temperature of the body was substituted. A constant flow of water into the bladder was kept up for three days, when the catheter was withdrawn and the urine examined, which, on previous examinations, was alkaline, but now, for the first time, was acid. Irrigation at intervals, varying from two to three days, was kept up for about one month, at the end of which time the case was discharged cured. Case two was not unlike the first, only in duration of time; about one month of treatment, by constant irrigation, at intervals varying as about in case one, was sufficient to cure the patient. W. F. L.

*Treatment of Wounds.* PROF. V. DUMREICHER. (*Wiener Mediz. Wochenschrift.* XXVII—6.)

The rapid healing of wounds without any, or but very slight fever, and without any, or but little secretion of pus; and how to prevent the occurrence of traumatic diseases—these problems have given rise to much discussion, and yet they cannot be considered as satisfactorily solved.

Lister has accomplished a great deal. Nussbaum, Volkmann, Thiersch and many others, have had very favorable results from Lister's method. They all attempt to prove, from their experiments, the superiority of Lister's antiseptic method over the common open treatment of wounds.

The advantages claimed for Lister's method are:

1st. The protection of wounds from all untoward influences, therefore preventing traumatic diseases.

2d. The more rapid healing of wounds, either *per primam intentionem*, without fever, or rapid healing, with very slight fever and but little suppuration.

After Volkman, Thiersch and Nussbaum made their report to the German Congress of Surgeons of the year 1876, recommending Lister's antiseptic treatment, Burrow reported on the success his father and himself have had in the open treatment of wounds.

The hospital under their supervision contained 16 beds; ventilation imperfect; the patients of the poorest class; several amputations and other operations were performed by students. Under these circumstances, their report shows that out of 123 operations but 7 per cent. proved fatal. While Listers shows a mortality of 17 per cent., Volkmann, 18 per cent., and Thiersch, 23 per cent.

The authors who are in favor of Lister's method differ in their opinions as to its protective power. Nussbaum looks upon it as a prophylactic agent against hospital gangrene rather than against pyæmia. Volkmann does not think that Lister's method will protect the patient entirely from traumatic diseases, particularly not from erysipelas; he, however, believes that these diseases will decrease in frequency and severity under its use. Thiersch believes that Lister's method is a prophylactic against pyæmia.

For the open treatment of wounds, as practised formerly, well-ventilated apartments were required, the greatest cleanliness was necessary, and an experienced corps of assistants and nurses was indispensable. The constant supervision of the surgeon was also required. Any of these conditions failing, the treatment was not a success.

In the technic of operative surgery decided progress has been made —improvements that were essential for the open treatment. We have the catgut ligature which causes no suppuration, and if the wound is closed, will be absorbed; anæsthetics and Esmarch's bandage allow the surgeon to prepare the wounded surfaces as he pleases.

A close study of Lister's method shows that the largest wounds, if properly attended to, can heal by first intention. Considerable experience is required to properly apply the bandages so as to obtain favorable results.

To fully carry out the directions given by Lister would require skilled assistants and costly bandages, and if not carried out strictly according to directions, our labor will be for naught. For a country surgeon, it would be almost impossible to make use of this method.

Prof. D. has his doubts as to whether the antiseptic measures, as laid down by Lister are at all necessary.

We know that in healthy individuals a wound will heal by first intention, if the edges of the wound are brought into close contact and held there; and that if the person is unhealthy, his blood impoverished, a similar wound will not heal by first intention, owing to a want of plasticity of the exudation between the surfaces.

A wound in a healthy person can be healed by first intention if the following conditions are fulfilled:

1st. To give the surfaces of the wound such a form that they can be brought in the closest contact.

This can be easily accomplished in operations of the extremities by using anæsthetics and Esmarch's bandage.

2nd. To use a ligature that will be absorbed and cause no suppuration.

3d. To cause the wounded surfaces to secrete a copious plastic exudation. This can be accomplished by applying a 4 to 8 per cent. solution of chloride of zinc to the surfaces.

4th. When there there is no longer any danger from hemorrhage, to bring the opposite surfaces in close contact.

5th. When it is impossible to bring the surfaces in close contact, to allow a free escape to any pus that may be formed. This we can do by using the drainage tube.

6th. To prevent the dressings, after being impregnated with the secretion of the wound, from adhering to the skin, because, in removing them, we may disturb the parts, by partly tearing open the wound. After the wound has been treated as above directed, a piece of waxed paper is placed over it, openings being made in the paper for drainage tubes, if any are used.

To cause the surfaces to come in closest contact, cotton is put over the surfaces of the wounds, and through adhesive plaster and bandages, the necessary compression is made. The cotton being elastic, there is no great danger of making too severe pressure.

This method was tried in cold abscesses, exudations in the sheaths of tendons and hydrocele, with the best of results.

The results have been so gratifying as to prove that Lister's good results are not due to the antiseptic measures adopted, but to the close adaptation of the surfaces, to the exciting of inflammatory exudations and to the free escape of pus. Dumreicher, therefore, cannot accept the doctrine of Lister, maintaining that the favorable course of all wounds treated according to his method and the absence of all traumatic diseases are due to his antiseptic measures.

Stich and Panum, by their experiments on animals, proved that pyæmia and septicæmia could only be produced by direct infusion of the poison into the veins. Burrow's statistics show that by cleanliness, poisoning of wounds can be prevented. During the operation, the escape of blood from veins prevents the introduction of poison; after the operation, the air, which carries the septic germs, is excluded by the close union of the edges. and the drainage tubes are rendered impervious to air by closing their external end.

D. considers it a rather one-sided view, which claims that traumatic diseases are produced mostly by external influences, air, etc.; he thinks that the individuality of the person, his state of health, the condition of his blood, etc., etc., are very important factors.

*How to Prevent Hemorrhage After the Removal of Esmarch's Bandage.* DR. RIEDINGER. (*Deutsche Zeitschrift fur Chirurgie*, XVII., 5 and 6.

The greatest disadvantage attached to the use of Esmarch's bandage, is the profuse hemorrhage following the removal of the elastic band. The hemorrhage in profuseness depends upon the force of constriction, and the length of time which it is allowed to remain. The amount of blood lost in many cases is often more than that following digital compression.

Esmarch himself admits that there is considerable capillary hemorrhage after the removal of his bandage, but thinks that it is easily stopped and of no long duration.

The author mentions the names of many eminent surgeons, who have seen the most profuse hemorrhage after severe operations, particularly after amputations; the blood pouring out of the wounded surfaces as if from a sponge.

Various surgeons of England, France and Germany are mentioned, all of whom have had unpleasant experiences with this secondary hemorrhage.

The author believes that hemorrhage is caused through paralysis of the vaso-motor nerves; further, that through the constriction, the blood being forced entirely from the part operated, no coagulation of the blood takes place.

Various means have been tried to check this hemorrhage, but they have, as a rule, not been very successful, *e. g.*, cold applications, ice, ice-water; but the hemorrhage persists in spite of these applications often fifteen minutes, and sometimes one-half to one hour.

Esmarch recommends the ligation of every blood vessel, veins as well as arteries.

P. Bruns has ligated as many as thirty-six vessels, and still had hemorrhage. Bardeleben has ligated all arteries mentioned in anatomy, and those vessels presenting themselves to the eye, arteries and veins, and still had considerable hemorrhage.

The author has performed experiments on animals to see whether the sensibility of the nerves was entirely overcome by the constriction of bandage. The reaction upon applying electricity was quite apparent, and from this he was led to apply electricity to the nerves which supply the vessels of the part operated on; his results were quite satisfactory, so much so that he recommends the application of electricity before the removal of the bandage. He uses the induced current. The poles to terminate in sponges; one pole to be placed on the wounded part, the other electrode to be passed over the nerve or nerves which distribute branches in the bandaged part, he has succeeded in reducing the hemorrhage to a great extent.

